# Physiological Comparison of Concentric and Eccentric Arm Cycling in Males and Females

**DOI:** 10.1371/journal.pone.0112079

**Published:** 2014-11-05

**Authors:** C. Martyn Beaven, Sarah J. Willis, Christian J. Cook, Hans-Christer Holmberg

**Affiliations:** 1 Swedish Winter Sports Research Centre, Department of Health Sciences, Mid Sweden University, Östersund, Sweden; 2 School of Sport, Health and Exercise Sciences, Bangor University, Bangor, United Kingdom; Universidad Europea de Madrid, Spain

## Abstract

Lower body eccentric exercise is well known to elicit high levels of muscular force with relatively low cardiovascular and metabolic strain. As a result, eccentric exercise has been successfully utilised as an adaptive stressor to improve lower body muscle function in populations ranging from the frail and debilitated, to highly-trained individuals. Here we investigate the metabolic, cardiorespiratory, and energy costs of upper body eccentric exercise in a healthy population. Seven men and seven women performed 4-min efforts of eccentric (ECC) or concentric (CON) arm cycling on a novel arm ergometer at workloads corresponding to 40, 60, and 80% of their peak workload as assessed in an incremental concentric trial. The heart rate, ventilation, cardiac output, respiratory exchange ratio, and blood lactate concentrations were all clearly greater in CON condition at all of the relative workloads (all *p*<0.003). Effect size calculations demonstrated that the magnitude of the differences in *V*O_2_ and work economy between the ECC and CON exercise ranged from very large to extremely large; however, in no case did mechanical efficiency (η_MECH_) differ between the conditions (all *p*>0.05). In contrast, delta efficiency (η_Δ_), as previously defined by Coyle and colleagues in 1992, demonstrated a sex difference (men>women; *p*<0.05). Sex differences were also apparent in arteriovenous oxygen difference and heart rate during CON. Here, we reinforce the high-force, low cost attributes of eccentric exercise which can be generalised to the muscles of the upper body. Upper body eccentric exercise is likely to form a useful adjunct in debilitative, rehabilitative, and adaptive clinical exercise programs; however, reports of a shift towards an oxidative phenotype should be taken into consideration by power athletes. We suggest delta efficiency as a sensitive measure of efficiency that allowed the identification of sex differences.

## Introduction

In 1892 Adolf Fick clearly demonstrated in thermometric studies on isolated muscle that more heat is produced when the muscle is actively stretched than during active shortening [1]. While in 1896, Chauveau reported that in human subjects the cost of descending stairs backwards (negative work or travail négatif) was substantially less than ascending them forwards (positive work or travail positif) [2]. Since this time, it has become abundantly clear that excentric (eccentric) exercise can be performed with a lower metabolic, ventilatory, and haemodynamic cost when compared to concentric exercise [3]–[10].

Consequently, lower limb eccentric training has been applied to populations for whom high-intensity training is contraindicated such as the elderly [11]–[13] and COPD patients [14]. Further, eccentric training generates extensive mechanical muscle tension [15] which is associated with activation of mitogen-activated protein kinases [16]. This property has led to the utilization of lower limb eccentric training in athletic populations to induce muscular overload and enhance maximal strength, muscular stiffness, and muscle accretion [15], [17], [18].

Unsurprisingly, using a novel arm ergometer fabricated in-house, our laboratory has previously confirmed that the low metabolic cost and high force generating capacities of eccentric contractions are not constrained to the muscles of the lower limbs. Specifically, we found that arm ergometry at three absolute power outputs (40, 80, and 120 W) performed at 60 revolutions min^−1^ were demonstrably and significantly less metabolically demanding (as assessed by oxygen uptake *V*O_2_) when performed eccentrically as compared to concentrically [19]. An acknowledged limitation of our previous study design is that the absolute workloads imposed may have taxed the participants differentially and influenced the observed responses to concentric and eccentric arm cycling. Herein, we address this shortcoming by prescribing relative workloads.

We were also interested in the effect of sex on the observed responses as our earlier study included only men. It is apparent that there exists an innate disparity in the relative strength and muscular endurance characteristics of men and women that are specific to the muscles of the upper body [20]. There are also known differences between upper and lower body oxygen kinetics [21], [22]. A relationship between cardiac output (Q _C_) and metabolic demand has been described in the lower limbs indicating that exercise-induced circulatory responses are mainly under metabolic control [9]; however, no such information is currently available for upper-body exercise.

In the present study, we tested the hypothesis that our previous observations of a significantly decreased metabolic cost of exercise at absolute work rates would be confirmed with relative work rates. Further, we hypothesized that sex differences in upper body muscle efficiency would be apparent. Lastly we sought to investigate the relationship between Q _C_ and metabolic demand in the muscles of the upper limbs.

## Methods

### Ethics Statement

The investigation was conducted according to the principles expressed in the Declaration of Helsinki. All participants provided informed written consent prior to participation and pre-approval was obtained from the Umeå Regional Ethical Review Board (# 2012-114-31M).

### Participants

Seven men and seven women (physical characteristics described in Table 1) volunteered to participate in this study. Participants were physically active in a variety of sports a minimum of three times per week. The protocol and procedures were explained verbally and all participants provided written informed consent prior to testing.

**Table 1:** Participant characteristics.

Variable	Group (n = 14)	Men (n = 7)	Women (n = 7)
Age (yr)	25.3±5.4	26.6±6.8	24.0±3.7
Height (cm)	174±9	179±9	169±6*
Body Mass (kg)^#^	74.2±9.6	82.0±7.5	67.5±5.0**
Lean Body Mass (kg)^#^	54.6±11.6	65.4±6.6	45.4±4.0**
Body Fat Mass (kg)^#^	16.3±4.3	12.9±2.3	19.2±3.4**
Body Fat Mass (%)^#^	23.5±7.6	16.5±2.9	29.5±4.0**
Arm Lean Mass (kg)^#^	6.8±2.2	8.9±0.9	5.0±0.7**
Arm Fat Mass (kg)^#^	1.9±0.5	1.5±0.2	2.2±0.3**
Arm Fat Mass (%)^#^	22.8±9.0	14.2±2.1	30.1±4.5**
*V*O_2PEAK_ (L·min^−1^)	2.7±0.6	3.0±0.6	2.3±0.4*
*V*O_2PEAK_ (mL·kg^−1^·min^−1^)	35.8±5.7	38.0±6.2	34.0±5.0
HR_PEAK_ (beats·min^−1^)	182±9	180±9	185±9
W_PEAK_ (W)	138±29	157±22	119±23**

All values are reported as mean ± standard deviation. *V*O_2PEAK_ Peak oxygen consumption, HR_PEAK_ Peak heart rate, W_PEAK_ Peak power production. All peak values refer to those obtained in an incremental concentric arm cycling test (60+15 W·60 s). ^#^ As assessed by dual-energy X-ray absorptiometry (GE, Lunar iDXA). *p<0.05, **p<0.01.

### Experimental Protocol

The week before the experimental data collection, participants performed three sessions of eccentric (ECC) and concentric (CON) arm cycling in order to become familiar with the arm cycle ergometers and testing protocol. In addition, these practice trials enabled participants to acquire the specific coordination patterns required for eccentric arm cycling. It is important to point out that eccentric exercise to which an individual is unaccustomed is commonly associated with direct and indirect measures of reversible muscle damage as well as subjective increases in pain [23], [24]. To minimize the occurrence of such muscle soreness, the duration and intensity of the eccentric arm cycling trials were progressively increased over the three practice sessions (e.g. from 4 to 10 min and from 60 to 120 W). After this familiarization period, participants entered the experimental protocol only if they reported no residual muscle soreness as assessed using the perceived recovery status scale [25]. Following the three practice sessions, and at least 48 h prior to entering the experimental week, individual W_PEAK_ was determined from an incremental concentric arm cycling test that started at 60 W and increased by 15 W every 60 seconds.

During the experimental week, participants reported to the laboratory on two separate occasions to perform either eccentric or concentric arm cycling trials that were assigned in a random fashion. On each day, participants performed a concentric arm cycling warm-up for 5 min at 60 W. Subsequently, participants performed either eccentric or concentric arm cycling trials with 4-min efforts at 40, 60, and 80% of their individual W_PEAK_. Physiological responses were constantly measured as described below. A recovery period of 3.5 min was provided between each 4 min effort. All experimental visits were separated by at least 48 h and completed at the same time of day.

The eccentric and concentric arm cycle ergometers used in this investigation have been described in detail in a recent paper from our Swedish laboratory [19]. Briefly, an isokinetic eccentric arm cycle ergometer was constructed using a Monark 891E cycle ergometer frame, stand, and flywheel (Monark Exercise AB, Vansbro, Sweden). A 2.2 kW electric motor (BEVI 2SIE1004A, BEVI AB, Blomstermåla, Sweden) was connected to the flywheel via a pulley and a belt. Motor speed and pedaling rate were controlled by a variable frequency drive (Invertek Optidrive-E i55, Invertek Drives Ltd., Welshpool, UK). Cycling power was quantified using a power meter (Schoberer Rad Meßtechnik [SRM], Jülich, Germany), a system that has previously been shown to accurately quantify power output [26].

**Figure 1:**
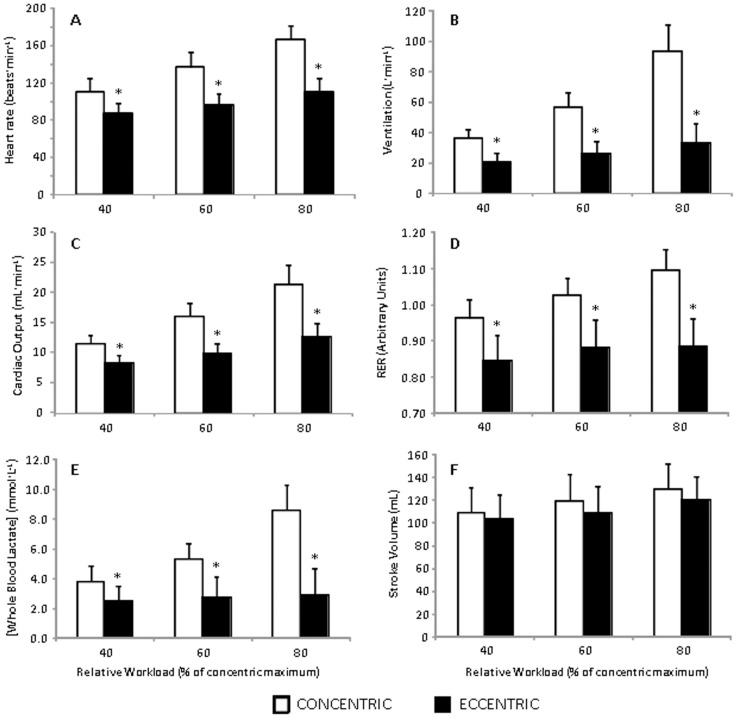
Metabolic and cardiorespiratory responses to eccentric and concentric arm cycling. Values are reported as mean ± standard deviation. *p<0.05 vs concentric exercise modality.

For the eccentric arm cycling trials, participants were instructed to resist the motor-driven handles of the ergometer at their individually specified target powers (i.e. 40, 60, or 80% of W_PEAK_). Pedaling rate was set at 60 revolutions min^−1^ and the SRM power meter (sampling at 1 Hz) displayed the power that the participant was absorbing. An illustration of the eccentric arm ergometer can be seen in Figure 1 of our previous article [19]. During each trial, participants were given at least 10 s to stabilize at this target power, which was then maintained for 4 min during which physiological responses were monitored and recorded.

The concentric arm cycling ergometer was fabricated by adapting a mechanically braked Monark 839E cycle ergometer. For the concentric arm cycling trials, participants were instructed to cycle at 60 revolutions min^−1^ whilst the ergometer (in constant power mode) maintained the prescribed power (i.e. 40, 60, or 80% of W_PEAK_). The SRM power meter (sampling at 1 Hz) displayed pedalling rate and the power produced by the participant.

Although the cycle ergometers differed for the eccentric and concentric arm cycling trials (i.e. constant pedalling rate vs. constant power) SRM crank power was measured at the same location in both cases. Both SRM power meters were calibrated using the same static procedures with the average calibration factor (Hz·N·m^−1^) entered into the SRM power control unit from four different positions [19], [27]. Finally, individual ergometer and seat positions were carefully adjusted and standardized such that the crank axle was located just below the level of the heart and the elbow positioned at a comfortable angle (∼20° between the ulnar notch and humoral head when the cranks were horizontal), since posture is known to influence force production capacity [28]. Both arm cycle ergometers were set up in an asynchronous configuration, with the pedal arms of the flywheel oriented at 180° relative to one another.

### Physiological Measures

Gas exchange values were measured with a mixed expired procedure using an ergospirometry system (AMIS 2001 model C; Innovision A/S, Odense, Denmark), equipped with an inspiratory flowmeter. The gas analyzers were calibrated with a high-precision mixture of 16.0% O_2_ and 4.5% CO_2_ (Air Liquide, Kungsängen, Sweden), and calibration of the flowmeter was performed at low, medium, and high flow rates with a 3-L air syringe (Hans Rudolph, Kansas City, MO). Ambient conditions were monitored with an external apparatus (Vaisala PTU 200; Vaisala Oy, Helsinki, Finland). Oxygen consumption (*V*O_2;_ L min^−1^), ventilation (V_E_; L min^−1^) and the respiratory exchange ratio (RER) were monitored continuously, and values were averaged over the final 30 s of each trial. Heart rate was recorded using a Polar monitor (Polar S610, Polar Electro Oy, Kempele, Finland). Overall rating of perceived exertion (RPE_BODY_) as well as arm-specific RPE (RPE_ARMS_) were assessed during the final 30 s of each trial using a Borg 6–20 scale [29]. Whole blood lactate (20 µL, Biosen 5140, EKF-diagnostic GmbH, Magdeburg, Germany) was collected from the fingertip within 1 min after each trial.

Stroke volume (SV; ml), cardiac output (*Q*_C_; mL min^−1^), arterial pressure (mmHg), and systemic vascular resistance (SVR; dyn·s·cm^−5^) were determined employing thoracic electrical bioimpedance utilizing Signal-Morphology Impedance Cardiology (SM-ICG) and High Definition Impedance (HD-Z) technologies (Physio Flow, Manatec type PF05L1, Paris, France) during the final 30 s of each trial. The PhysioFlow device emits high-frequency (75 kHz) and low-amperage (3.8 mA peak-to-peak) alternating electrical current via skin electrodes and calculates SV independently of baseline impedance signal (Z0) relying on changes in the impedance signal (ΔZ) that is independent of hydration status, the inter-electrode distance, and the resistivity of the blood [30]. A cardiac index (L min^−1^ m^−2^) was calculated from the *Q*_C_/BSA, where BSA is the body surface area (m^−2^) calculated using the Haycock method: BSA = 0.024265 x Height^0.3964^ x Weight^0.5378^.

Before each test, the bioimpedance device was calibrated according to the manufacturer's instructions. The participant’s skin was shaved and then abraded with a specialized skin preparation gel (Nuprep, Weaver and Co., Aurora, CO, USA), and cleaned with a 70% ethanol solution prior to the application of 6 self-adhesive AgCl electrodes (PhysioFlow, PF-50, Leonhard Lang GmbH, Innsbruck, Austria). The clinical acceptability of this bioimpedance device has previously been established against the direct Fick method over a wide range of Q _C_ values (3.55 to 26.75 L min^−1^) during repeated incremental exercise separated by three days in healthy subjects [31], [32]. Bioimpedance cardiography has also been demonstrated to exhibit superior reproducibility (as estimated from the variability between repeated measures) compared to other non-invasive methods of cardiac output estimation [33]. Mean arterial pressure was calculated as [(2 × diastolic arterial pressure)+systolic arterial pressure]/3 [9]. The arteriovenous oxygen difference [(A-V)O_2_] was was assessed indirectly via the Fick Principle: *V*O_2_ = *Q*_C_×(C_a_O_2_–C_v_O_2_), where C_a_O_2_ is the arterial oxygen content and C_v_O_2_ is the venous oxygen content.

Work economy (W L^−1^ min^−1^) as well as both gross mechanical efficiency (η_MECH_) and delta efficiency (η_Δ_) were calculated. η_Δ_ was assessed as it eliminates the influence of metabolic processes that do not contribute to the work performed and thus has been suggested as a more valid indicator of muscular efficiency [34], [35]. Specifically, work economy was calculated as the mechanical work performed per litre of oxygen consumed in one minute. η_MECH_ (%) during sub-maximal steady-state was calculated by dividing the internally liberated metabolic power by the external power output as described by Hopman and colleagues [36]: η_MECH_ = W/(*V*O_2_ × 340), where W represents the internally liberated mechanical power and 340 represents the power equivalent of 1 L of O_2_ min^−1^. Finally, the η_Δ_ (%) was calculated from the reciprocal of the slope of the linear regression line that described the relationship between energy expenditure and work rate [34].

### Data Analysis

Requisite transformations of the efficiency (arcsineroot transformation) and physiological (log transformation) data was performed prior to statistical analysis to reduce bias arising from non-uniformity of error (Hopkins et al. 2009). Separate analyses of variance procedures were performed to assess differences in physiological responses between the CON and ECC conditions. Subsequent paired student's t-tests were performed on relevant comparisons. The magnitude of between-condition differences in the means were expressed as an effect size (ES), which were calculated using the pooled standard deviations [37]. Threshold values for ES statistics were >0.2 (small), >0.6 (moderate), >1.2 (large), >2.0 (very large) and >4.0 (extremely large). Confidence intervals (90%) for the (true) mean changes or between-group differences were estimated (Hopkins et al. 2009). Quantitative chances of the likelihood of between condition differences were assessed qualitatively as follows: ≤1% almost certainly not, >1–5% very unlikely, >5–25% unlikely, >25–75% possible, >75–95% likely, >95–99 very likely, >99% almost certain. The effect was deemed ‘clear’ if its confidence interval did not overlap the thresholds for small positive and negative effects [38]. Bi-variate relationships between variables of interest were examined via multiple regression to control for between subject variation (r). Magnitudes of correlations were interpreted using thresholds of 0.1, 0.3, 0.5, 0.7 and 0.9 for small-, moderate-, large-, very large- and nearly perfect correlations respectively. Significance was set at an alpha level of *p*≤0.05.

## Results

The incremental concentric arm cycling test established work loads of 55±11, 84±18 and 111±23 W corresponding to 40, 60 and 80% of W_PEAK_. The actual work performed in the three CON and ECC trials was 40.5±1.5, 60.6±1.0, and 80.2±1.0 and 40.6±1.2, 60.3±1.4, and 78.8±1.3% of W_MAX_, respectively, demonstrating excellent agreement with the prescribed workloads. Notably, the 120-W absolute workload imposed in our prior work corresponded to a relative workload that ranged from 65 to 99% of W_PEAK_ in the current male cohort; thus reinforcing the importance of prescribing relative work when assessing physiological load in this type of experiment. Also note that for clarity and ease of comparison, all power values measured during eccentric cycling are expressed as absolute values herein. The average pre-trial rating on the perceived recovery status scale was 7.9±1.6 corresponding to nominal values associated with an expectation of similar performance [25].

When compared with the ECC condition, the HR responses (all *p*<0.0001), V_E_ (all *p*<0.0001), *Q*_C_ (all *p*<0.0001), RER (all *p*<0.0001) and blood lactate concentration (all *p*<0.003) were all almost certainly higher in the CON condition at all three workloads (Figure 1A to 1E). RPE_BODY_ (all *p*<0.016) and RPE_ARMS_ (all *p*<0.002) were also clearly elevated in the CON compared to the ECC condition. There was no clear difference in SV between the conditions (Figure 1F). We also noted a near perfect correlation between the HR obtained from the PhysioFlow and Polar monitors (r = 0.99).

**Figure 2:**
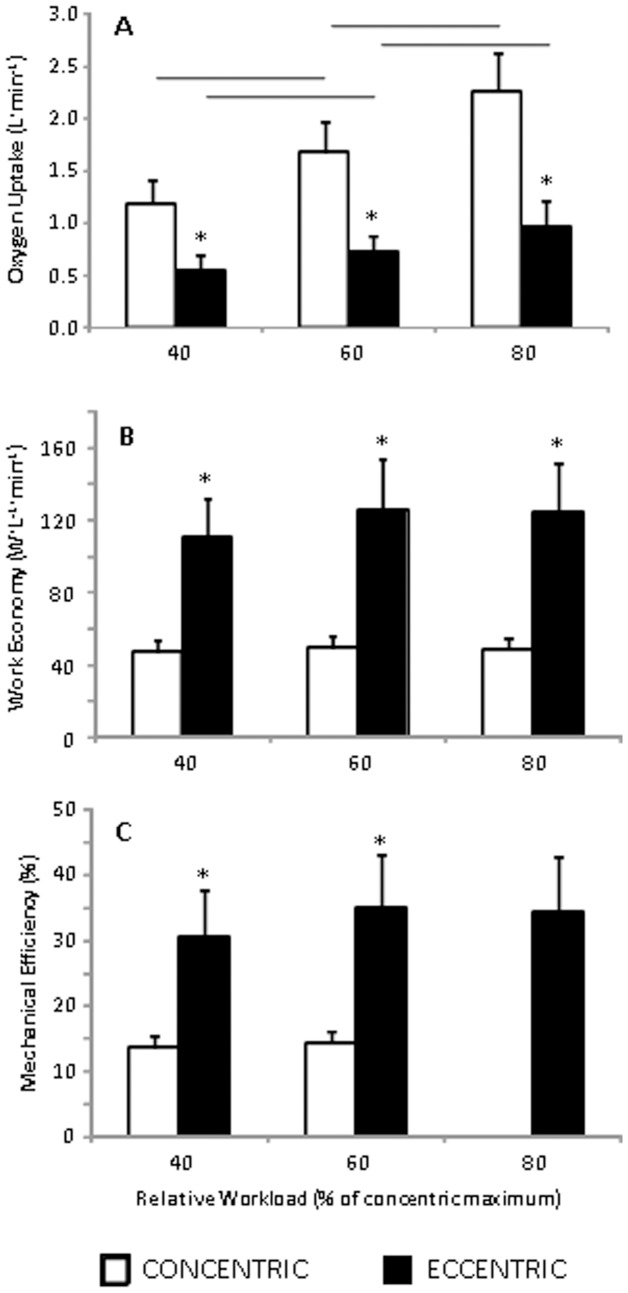
Energy cost of eccentric and concentric arm cycling. Values are reported as mean ± standard deviation. *p<0.05 vs concentric exercise modality. Horizontal bars represent significant differences between the indicated workloads *p*<0.05.

**Figure 3:**
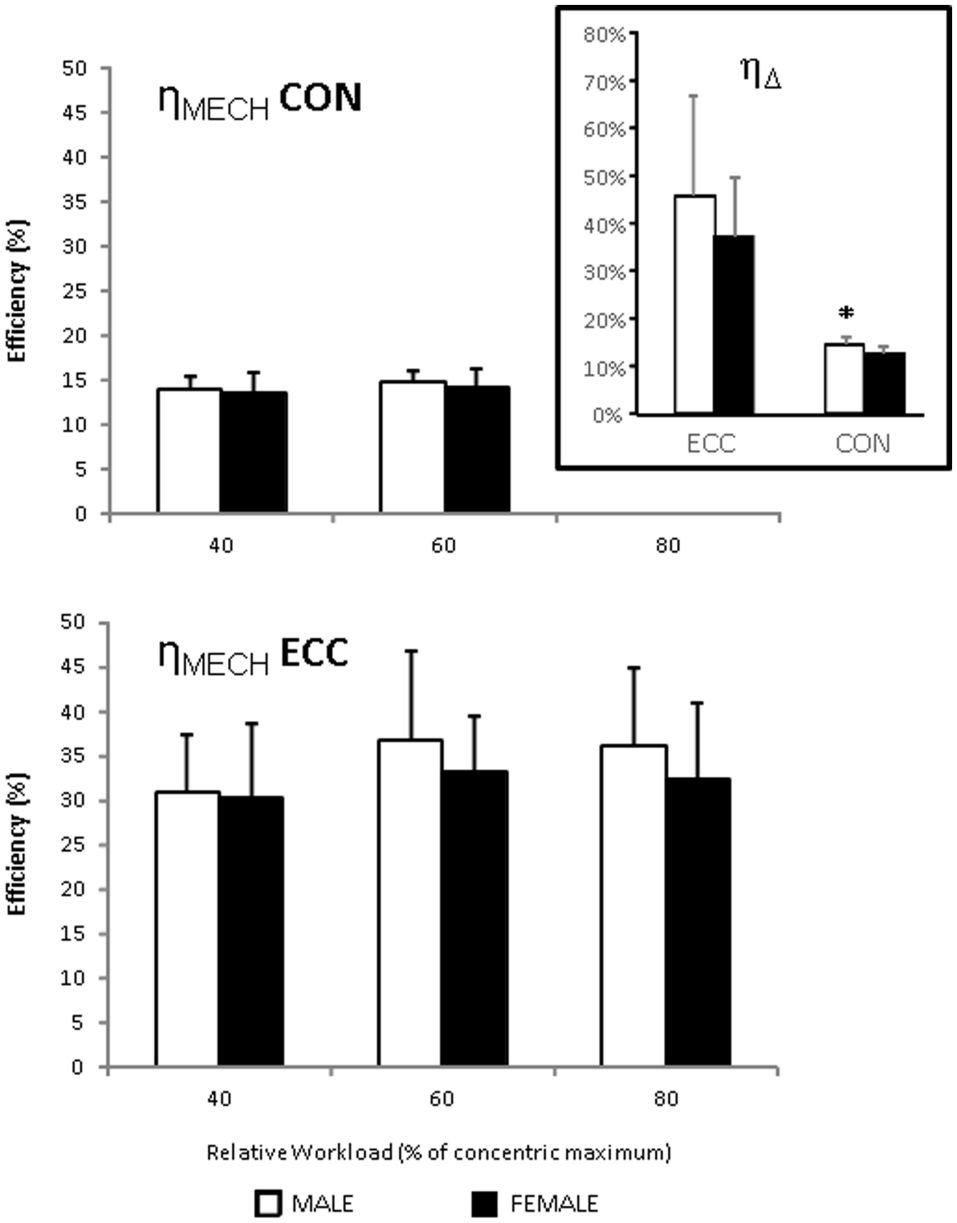
Sex differences in efficiency measures of eccentric and concentric arm cycling. Values are reported as mean ± standard deviation. *clear difference vs male participants.

Extremely large or very large differences in *V*O_2_ were observed between the CON and ECC conditions at 40 (ES: 3.62±0.63; *p*<0.0001), 60 (ES: 4.16±0.63; *p*<0.0001), and 80% of W_PEAK_ (ES: 4.08±0.63; *p*<0.0001; Figure 2A). *V*O_2_ consistently increased as workload increased in both conditions (all *p*<0.004). The qualitative magnitudes of the differences in economy (all ES >3.83), and η_MECH_ (all ES >3.83) between the CON and ECC conditions were either very large or extremely large (Figure 2B & 2C). In no case did η_MECH_ differ at the different workloads (all *p*>0.05; Figure 2C). It was noteworthy that a clear sex difference of 1.7±1.7% (ES: 0.95) in concentric η_Δ_ was observed (men>women), that was not apparent in the η_MECH_ metrics (Figure 3).

**Figure 4:**
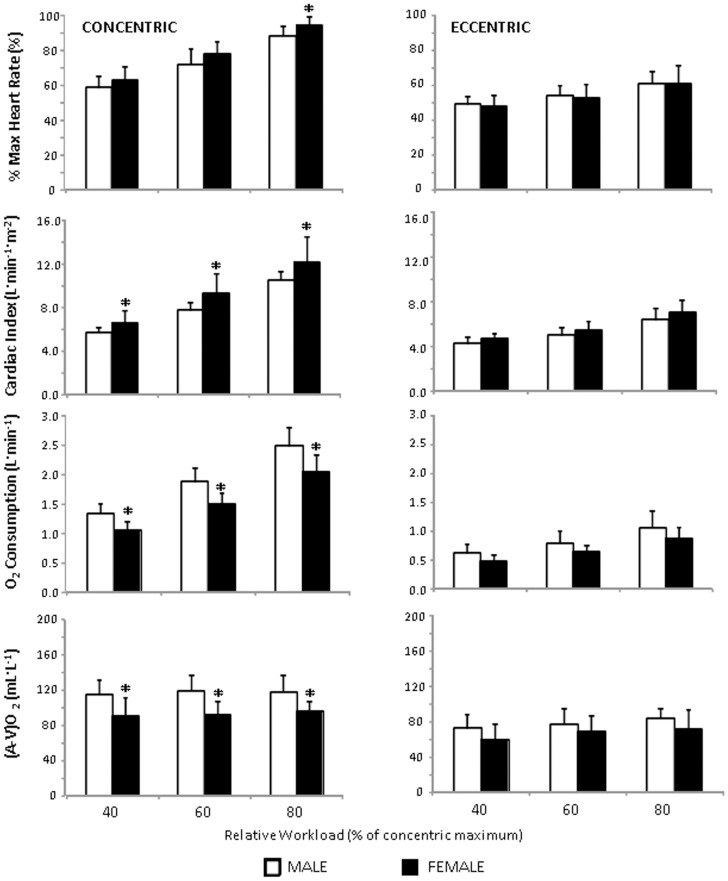
Sex differences in cardiovascular measures of eccentric and concentric cycling. Values are reported as mean ± standard deviation. *clear difference vs male participants. (A-V)O_2_, arteriovenous difference.

While no sex differences were apparent in *Q*_C_ (all p>0.5), the HR was consistently and substantially higher in females by 10 to 16 bpm during concentric exercise (ES 0.71 to 1.23) which corresponded to a 4.2 to 6.4% higher percentage of the maximum heart rate observed in the incremental concentric arm cycling test (Figure 4A). The cardiac index was also higher in women at all concentric workloads by 0.85 to 1.62 L min^−1^ m^−2^ (ES 0.84 to 1.12; Figure 4B). Interestingly, despite the relative loads prescribed, *V*O_2_ during concentric exercise was lower in women at all workloads by 0.30 to 0.43 L min^−1^ (p≤0.0172; ES: 1.40 to 1.83; Figure 4C) and thus the internally liberated metabolic power of concentric arm cycling was also lower in women (all p≤0.0182; ES: 1.49 to 1.95). A corollary of the fact that *V*O_2_ was lower in women, despite no difference between the sexes for *Q*_C_, was that the (A-V)O_2_ was of lesser magnitude in women (all p≤0.0340; Figure 4D). Overall, the (A-V)O_2_ was greater during concentric than eccentric exercise at 40% (ES: 1.98±0.65), 60% (ES: 1.62±0.65), and 80% (ES: 1.45±0.65) of W_PEAK_ (all p≤0.001). In both exercise conditions, the (A-V)O_2_ tended to increase with workload (CON: 102 to 106 ml O_2_·L^−1^, 4.2±4.1%, p = 0.0831; ECC: 66 to 78 ml O_2_·L^−1^, 15.4±8.3%, p = 0.0028).

**Figure 5:**
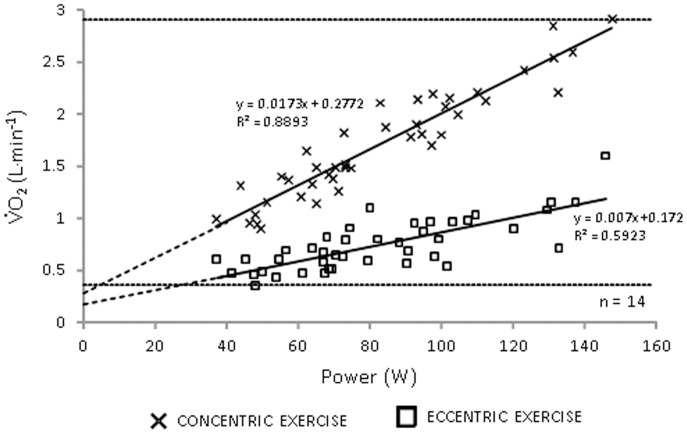
Absolute power output and oxygen consumption for eccentric and concentric arm cycling. Data represents all 14 individuals assessed across three relative workloads.

**Figure 6:**
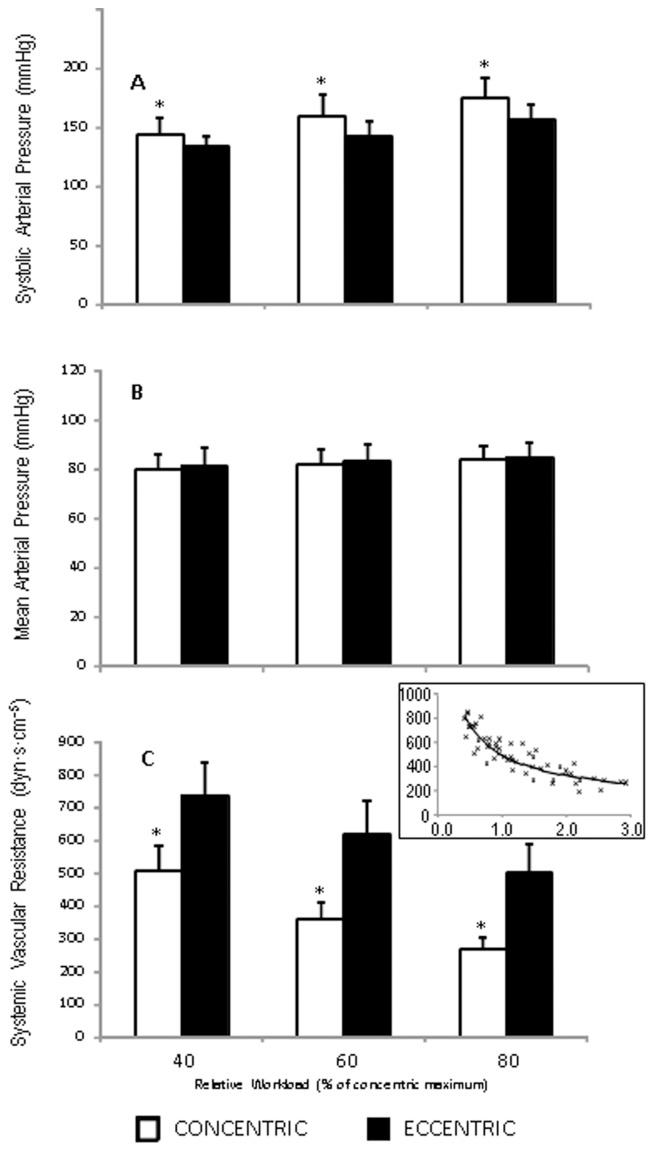
Arterial pressure and systemic vascular resistance plots responses to eccentric and concentric arm cycling. Values are reported as mean ± standard deviation. *p<0.05 vs concentric exercise modality. A: Systolic Arterial Pressure; B: Mean Arterial Pressure; C: Systemic Vascular Resistance; Inset: relationship between VO2 (x-axis: L min^−1^) and Systemic Vascular Resistance (y-axis: dyn·s·cm^−5^).

Using multiple regression to control for between subject variation, *V*O_2_ was significantly related to *Q*_C_ (R^2^ = 0.84; *p*<0.0001) and HR (R^2^ = 0.87; *p*<0.0001), but not SV (R^2^ = 0.31; *p* = 0.24). Plotting *V*O_2_ against absolute work output resulted in distinct slopes for the CON (0.0173) and ECC (0.007) conditions (Figure 5). These linear relationships revealed that the oxygen requirement was 206% greater in the CON compared to ECC condition at the wattage corresponding to the lowest *V*O_2_ observed (26.8 W; 0.36 L min^−1^), a value that increased to 235% at the highest *V*O_2_ observed (152.2 W; 2.91 L min^−1^). When individual plots of *V*O_2_ against absolute work output were plotted the slopes for the CON (0.0198; range: 0.0158 to 0.0245) and ECC conditions (0.008; range 0.004 to 0.0160) were similar to the grouped data (Figure 5). Finally, it was apparent that, despite higher systolic arterial pressure in the CON trials (all *p*<0.0015; Figure 6A), the mean arterial pressure was not different between the conditions (all *p*>0.4; Figure 6B). This observation was explained by greater systemic vascular resistance in the ECC condition compared to the CON condition at all workloads (all ES >2.50; all *p*<0.0001; Figure 6C) with a decrease in vascular resistance as workload (*V*O_2_) increased (Figure 6 inset).

## Discussion

Our data clearly demonstrate the magnitude of the differences in metabolic and cardiovascular responses, and efficiency between eccentric and concentric arm cycling, extending previous observations in the lower-limbs. Thus, our hypothesis that eccentric arm cycling at the same *relative* workload is substantially less physiologically taxing than concentric arm cycling was confirmed. Further, we demonstrate that delta efficiency was sensitive enough to allow the detection of sex differences and thus suggest a greater relevance of delta efficiency, as compared to gross mechanical efficiency, as a measure of muscular efficiency. Together, these results emphasize the high-force, low-cost nature of multi-joint eccentric muscle actions and indicate that eccentric arm cycling may serve as a unique modality for training muscles in the upper body across a range of populations.

Our results document physiological responses to eccentric arm cycling that are in general agreement with those previously reported during eccentric leg cycling, even with considerable differences in muscle mass. Three key similarities can be identified that highlight the consistent nature of submaximal multi-joint eccentric muscle actions. First, *V*O_2_ during eccentric arm cycling was only a fraction of that during concentric arm cycling performed at an equivalent relative workload (Figure 2A) with the eccentric cycling being relatively more metabolically efficient at higher loads as suggested previously [8]. These extremely large differences in *V*O_2_ are consistent with the early work of Abbott and colleagues [39] and others [8]–[10] who have demonstrated that *V*O_2_ during eccentric leg cycling is ∼1/7 to 1/2 of that during concentric leg cycling. The reduced oxygen demand during eccentric exercise has been attributed to lower muscle activation or may result from the active muscle fibers consuming less oxygen [3], [9], [10].

Secondly, at a similar metabolic demand, the power produced during eccentric arm cycling was 2 to 3 fold greater than during concentric trials (Figure 4). These values are in agreement with previous reports that power absorption during eccentric leg cycling can be as much as five times greater than power production during concentric leg cycling [9], [10]. Thus, both lower- and upper-body eccentric cycling can facilitate mechanical overloading of muscle groups to a greater extent than concentric cycling, which has important ramifications for increasing muscle mass and strength [16], [40].

Thirdly, the exercise intensity during eccentric arm cycling was well below the blood lactate threshold of 4 mmol·L^−1^ (2.5 to 2.9 mmol·L^−1^) and whole-body and arm-specific perceived exertion ratings was low (6 to 12 on the Borg scale). Consequently, the novel aspect of eccentric arm cycling is that only low to moderate levels of perceived exertion are required to generate relatively high work rates, which lends this exercise modality to use for counteracting sarcopenia and maintaining muscle mass in populations that would otherwise be contraindicated (e.g. the elderly or COPD patients). These features have been well documented in eccentric leg cycling, demonstrating that the distinct physiological responses to submaximal multi-joint eccentric muscle actions are rather consistent for different muscle groups.

The distinct economy, η_MECH_ and η_Δ_ parameters of the eccentric contractions in the current study agree with the body of literature describing the superior efficiency of eccentric versus concentric contractions [6], [41]–[45]. η_Δ_ has been suggested to possess “conceptual advantages” over η_MECH_ in that the former is independent of metabolic processes that do not contribute to the work accomplished and therefore may provide a more valid measure of muscular efficiency [34], [35], [46]. We report a clear sex difference in η_Δ_ during CON contractions that was not apparent in η_MECH_. While, greater muscular endurance in the elbow flexors of women has previously been reported, which is suggestive of greater muscular efficiency [20], only one previous study has investigated η_Δ_ in arm cycling efficiency and found no difference between men and women [47]. However, these authors did not use a magnitude-based approach to identify differences and Figure 2b from their 2008 paper is suggestive of a difference in the mean in the order of one standard deviation when assessing work rates between 70 and 85% of individually prescribed ventilatory threshold putatively similar to that observed herein.

Other sex differences of note include the elevated heart rate and decreased (A-V)O_2_ apparent in the women during concentric exercise. In a study designed to investigate the difference between upper and lower body oxygen extraction, Calbet and colleagues [21] demonstrated that arm oxygen extraction was less efficient than in the muscles of the lower limbs. Similarly, Jensen-Urstad and Ahlborg [22] reported average (A-V)O_2_ between leg exercise (170 ml O_2_·L^−1^) and arm exercise (143 ml O_2_·L^−1^) preformed at 80% of *V*O_2MAX_, suggestive of the validity of the current measures (range 85 to 138 ml O_2_·L^−1^ at 80% of W_PEAK_). This prior research posited that lesser arm oxygen extraction capacity could be attributed to: a lower mass-normalized arm capillary muscle oxygen conductance; a lower diffusional area combined with a greater diffusional distance and; an inferior ability to counteract the exercise-induced vasodilatory response. Sex differences in these three physiological aspects could potentially explain the decreased efficiency observed in the current study. The markedly elevated absolute and relative heart rate and lower oxygen extraction during concentric exercise by the women in the current study provide evidence of a greater cardiovascular strain when maintaining a given workload and this was further evidenced by the disparity in cardiac output relative to body surface area (CI).

In an elegant assessment of the metabolic and mechanical factors that contribute to circulatory control, Dufour and colleagues [9] found that Q _C_ was primarily under metabolic control, demonstrating significant relationships between *V*O_2_ and *Q*_C_ (R^2^ = 0.75), HR (R^2^ = 0.84) and SV (R^2^ = 0.50) in the lower body. Here, we report similar relationships in the upper body between *V*O_2_ and *Q*_C_ (R^2^ = 0.84) and HR (R^2^ = 0.87) but not SV (R^2^ = 0.31). It is known that vascular reactivity differs between the arms and the legs, with greater changes in blood flow in response to physiological vasodilatory stimuli in the arms [48]. Indeed, the lower capacity of the arms to extract O_2_ from the blood implies that a greater cardiovascular effort is required to maintain a given metabolic rate in the upper limbs [21]. The relatively high systemic vascular resistance observed during our ECC condition may reflect an enhanced sympathetic drive, with decoupling of functional sympatholysis from the low metabolic demand [49]. “Differential circulatory adjustments” to muscle contraction type have been suggested to be related to muscle mechanoreceptor activation [50], and/or may simply reflect the lower demand for oxygen. Clinicians should be cognizant of the elevated vascular resistance when applying eccentric workloads in populations where raised arterial pressure would be contraindicated.

While there is little dispute as to the benefit of eccentric loading on muscle hypertrophy and strength measures [18], [51], [52], it is worth considering the possible implications of upper-body ergometry in athletic populations. Specifically, gains associated with eccentric training are highly specific to the velocity of movement and this may “compromise the transferability of strength gains to more functional movements” [40]. Such an assertion is supported by Cook et al. [18] who demonstrated “relatively negative” running sprint speed adaptations in well trained athletes exposed to an eccentric training program. Indeed, eccentrically biased contractions have been associated with specific adaptations which reflect a more oxidative muscle phenotype [12], [53], [54]. Interestingly, fast contractions (180° s^−1^) have been shown to activate proteolytic pathways, whereas slow contractions (30° s^−1^) lead to an upregulation of protein synthesis [55]. Indeed, Chapman and colleagues [56] demonstrated that, with equal time-under-tension, fast eccentric contractions (210° s^−1^) elicit more pronounced muscle damage than slow contractions (30° s^−1^). Collectively, these data suggest that the inclusion of high velocity eccentric contractions during training to more closely mimic dynamic actions common in sports should be carefully considered based on the desired outcomes.

In summary, the participants in this investigation performed repetitive multi-joint, eccentric upper-body exercise at substantially lower levels of metabolic, cardiovascular and ventilatory demand and perceived exertion than was associated with traditional concentric arm cycling. These data reinforce the distinct physiological responses to eccentric exercise and extend the application of lower body eccentric cycling to the muscles of the upper body. It is noted that delta efficiency (η_Δ_) allowed the identification of sex differences with men demonstrating a greater muscular efficiency. These findings suggest clear applications for upper body eccentric exercise in both debilitative and rehabilitative environments. Upper body eccentric exercise is also likely to form a useful adjunct for athlete training programs although the possible impact on dynamic actions highlighted by a shift to a more oxidative phenotype should be taken into consideration in the case of power athletes.
